# Chromosomal Analysis of Couples with Repeated Spontaneous Abortions in Northeastern Iran

**DOI:** 10.22074/ijfs.2015.4208

**Published:** 2015-04-21

**Authors:** Saeedeh Ghazaey, Fatemeh Keify, Farzaneh Mirzaei, Masumeh Maleki, Semiramis Tootian, Mitra Ahadian, Mohammad Reza Abbaszadegan

**Affiliations:** 1 Medical Genetics Research Center, Medical School, Mashhad University of Medical Sciences, Mashhad, Iran; 2 Pardis Clinical and Genetics Laboratory, Mashhad, Iran; 3 Division of Human Genetics, Immunology Research Center, Avicenna Research Institute, Mashhad University of Medical Sciences, Mashhad, Iran

**Keywords:** Chromosomal Abnormalities, Abortions, Cytogenetic Analysis

## Abstract

**Background:**

Cytogenetic study of reproductive wastage is an important aspect in determining the genetic background of early embryogenesis. Approximately 15 to 20% of all pregnancies in humans are terminated as recurrent spontaneous abortions (RSAs). The aim of this study was to detect chromosome abnormalities in couples with RSAs and to compare our results with those reported previously.

**Materials and Methods:**

In this retrospective study, the pattern of chromosomal aberrations was evaluated during a six-year period from 2005 to 2011. The population under study was 728 couples who attended genetic counseling services for their RSAs at Pardis Clinical and Genetics Laboratory, Mashhad, Iran.

**Results:**

In this study, about 11.7% of couples were carriers of chromosomal aberrations. The majority of abnormalities were found in couples with history of abortion, without stillbirth or livebirth. Balanced reciprocal translocations, Robertsonian translocations, inversions and sex chromosome aneuploidy were seen in these cases. Balanced reciprocal translocations were the most frequent chromosomal anomalies (62.7%) detected in current study.

**Conclusion:**

These findings suggest that chromosomal abnormalities can be one of the important causes of RSAs. In addition, cytogenetic study of families who experienced RSAs may prevent unnecessary treatment if RSA are caused by chromosomal abnormalities. The results of cytogenetic studies of RSA cases will provide a standard protocol for the genetic counselors in order to follow up and to help these families.

## Introduction

Approximately 15 to 20% of all pregnancies in humans result in recurrent spontaneous abortions (RSAs) (1). There are different reasons for RSAs including genetic abnormalities, maternal and paternal age, endocrine dysfunction, autoimmune disorders, infectious diseases, environmental toxins and congenital or structural uterine anomalies (2). Chromosomal unbalance have important role in abnormal early human development. Nearly, 50 to 60% of first-trimester spontaneous miscarriages have abnormal karyotype (3). Although the frequency of chromosomal abnormalities in couples with RSA varies between populations, it has been found higher frequency in the general population (0.3-0.4%) (4,5). Therefore, cytogenetic study of the parent with history of RSAs is an integral part of diagnostic clarification. Several cytogenetic 48 investigations have been performed in various countries to determine the pattern of chromosome abnormalities in parents with fetal wastage. The studies revealed that the prevalence of chromosomal anomalies varies from 2 to 8% in couples who are affected by RSAs (6). Unequal crossing over during meiosis can lead to chromosomal rearrangements producing gametes with unbalanced chromosomal aberrations like duplications or deletions, therefore, structural chromosome abnormalities in parents can be the major cause of recurrent miscarriages (7). The clinical outcomes of such unbalances generally are lethal to the developing embryo, leading to RSAs or early neonatal deaths (8). 

The objective of the current study was to determine the prevalence and types of chromosomal anomalies in couples living in Northeast of Iran, to compare our findings with those reported previously and to increase the awareness of physicians and gynecologists about the frequency and nature of chromosomal aberrations that contribute to recurrent miscarriages. 

## Materials and Methods

This retrospective study done over a 6-year period from 2005 to 2011 included 728 couples with history of abortions ranged 1-7 who were referred to the Genetic Counseling Services in Pardis Clinical and Genetics Laboratory (PCGL), Mashhad, Iran. All patients gave a signed informed consent and the study was approved by Ethics Committee of PCGL. 

All the referred couples were thoroughly examined, and detailed clinical and obstetric histories were recorded in prepared forms. The age of the couples, number of RSA and the possible existence of other causes for the abortion such as uterine malformations, hormonal insufficiency, and previously induced abortion(s) were investigated. 

For conventional cytogenetic study, 5 ml peripheral blood from each subject was collected into heparinized test tubes. Lymphocyte cultures were initiated according to Moorhead et al. (9). Next 400 µl whole blood cells were cultured in 5 ml RPMI 1640 medium (Gibco, USA), supplemented with 20% (v/v) fetal bovine serum (FBS, Gibco, USA) and 10 μg/ml phytohaemagglutinin (Gibco, USA) at 37˚C for 72 hours. Cultured cells were harvested by adding colcemid (Gibco, USA) for 10 minutes followed by treatment of hypotonic solution (0.075 M KCl, Merck, Germany) for 15 minutes, and the treated cells were then fixed using Carnoy’s fixative (3:1 methanol-glacial acetic acid; Merck, Germany). The karyotype of the couples was prepared using G-banding technique with trypsin and Giemsa staining (GTG) (10) and C-banding technique with barium hydroxide (11). Images of well-banded metaphases were obtained using olympus photomicroscope (BX-40, Japan) and were analyzed by CytoVision software (Applied Imaging, USA) at 400-550 band resolution. Karyotyping of 30 metaphases was performed routinely, while in cases of mosaicism, 100 metaphase spreads were analyzed. Karyotyping of each couple was carried out according to the International System for Human Cytogenetics Nomenclature (ISCN) 2009 (12). 

## Results

Couples’ ages ranged from 18 to 45, with a mean of 29.6. As mentioned in table 1, 11.7% showed abnormal karyotype. In 728 couples, we determined that 48 women and 37 men had chromosomal aberrations. Among chromosomal abnormalities, 52 structural and 7 numerical anomalies were detected. In addition, there were 27 cases with three types of polymorphic variants including constitutional fragility of chromosome 16, pericentric inversion of chromosome 9, and prominent satellites in chromosomes 13 and 15. There were two instances where both members of a couple had an abnormal karyotype (couples no.: 11, 12, 17, and 18, mentioned in Table 1).

Table 2 shows the distribution of couples according to the number of spontaneous abortions. As mentioned, 51.6% had two miscarriages and 14.6% had only one miscarriage. The remaining (33.8%) had 3 or more miscarriages. The structural chromosomal abnormalities we encountered were divided into balanced reciprocal chromosomal translocations (37/85), Robertsonian translocation (8/85) and inversions (7/85). Reciprocal translocations were the most prevalent abnormality. Inversions, marker chromosomes and Robertsonian translocations were seen with a trend of decreasing percentage, respectively. The highest percentage of chromosomal aberration was seen in couples with five or more RSAs.

**Table 1:** Cytogenetic study, number of abortions, and parental age in cases with structural abnormalities


	Karyotypes	No of cases	Age	No of abortions

	Robertsonian translocations			
**1**	45,XY,t(15;15)(q10;q10)	1	27	2
**2**	45,XX,t(13;14)(q10;q10)	2	30, 35	2, 2
**3**	45,XY,t(13;14)(q10;q10)	3	22, 25, 37	2, 2, 3
**4**	45,XX,t(14;15)(q10;q10)	2	27, 32	2, 3
	Reciprocal translocations			
**1**	46,XX,t(2;15)(q25;q26.1)	1	28	2
**2**	46,XX,t(3;6)(q29;p21.1)	1	31	3
**3**	46,XX,t(1;3)(q22.2;q25.2)	1	27	2
**4**	46,XY,t(7;18)(p21.3;q12.2)	1	41	4
**5**	46,XX,t(4;7)(q34.3;q21.3)	2	37, 42	4, 5
**6**	46,XX,t(7;14)(q36;q24.3)	1	24	3
**7**	46,XX,t(12;22)(q10;q10)	2	30, 33	3, 4
**8**	46,XY,t(12;22)(p11.2;p11.2)	1	34	2
**9**	46,XY,t(6;10)(p25;p11.2)	1	36	3
**10**	46,XX,t(10;21)(p21.1;q22.2)	1	25	2
**11**	46,XY,t(6;16)(q26;p12)	1	34	4
**12**	46,XX,t(6;16)(q26;p12)	1	26	4
**13**	46,XY,t(9;17)(q22.1;p13.1)	1	26	2
**14**	46,XY,t(4;20)(q32;p12)	1	34	3
**15**	46,XX,t(8;17)(q24.3;q21)	1	27	2
**16**	46,XX,t(3;7)(q22;q32)	1	34	3
**17**	46,XY,t(11;22)(q23;q11)	2	29, 39	2, 5
**18**	46,XX,t(11;22)(q23;q11)	1	26	2
**19**	46,XX,t(15;20)(p10;p10)	1	29	2
**20**	46,XY,t(8;11)(p23;q21)	1	45	5
**21**	46,XX,t(8;11)(p23;q21)	1	27	4
**22**	46,XY,t(16;22)(q23;q12)	1	27	2
**23**	46,XX,t(2;18)(p21;q11.2)	1	24	2
**24**	46,XX,t(8;10)(q13;q22.2)	1	31	3
**25**	46,XX,t(13;20)(q22;p13)	2	18, 22	1, 2
**26**	46,XY,t(4;5)(q25;p15.2)	1	42	4
**27**	46,XX,t(5;6)(q34;p21.2)	1	27	2
**28**	46,XX,t(2;7)(q34;q34)	1	29	3
**29**	46,XX,t(2;7)(q37.1;q32)	1	37	4
**30**	46,XY,t(6;8)(p23;q12.2)	1	40	5
**31**	46,XX,t(10;12)(q23.2;q21.3)	1	29	1
**32**	46,XY,t(4;6)(q23;q21)	1	35	3
**33**	46,XX,t(10;17)(p13;q21.3)	1	36	7
	Pericentric inversions			
**1**	46,XX,inv(5)(p15.3q15)	2	27, 37	2, 2
**2**	46,XY,inv(10)(p14q21)	2	30, 33	2, 2
**3**	46,X,inv(Y)(p11.2q11.22)	3	24, 33, 41	2, 2, 4
	Numerical abnormalities			
**1**	47,XYY	1	41	4
**2**	47,XXY/46,XY	2	27, 36	1, 2
**3**	45,X/46,XX/47,XXX	3	22, 24, 32	2, 2, 3
**4**	47,XXX	1	31	2
	Polymorphic variants			
**1**	46,XY,inv(9)(p11q13)	11	18, 36	2, 5
**2**	46,XX,inv(9)(p11q13)	9	20, 42	2, 7
**3**	46,XX, Frag16q21	2	25, 31	2, 3
**4**	46,XX/46,XX, Frag 16q21	1	28	2
	13p^+^	2	28, 31	1, 2
	15p^+^	1	20	1
	Total	85 (11.7%)		


t; Translocation, inv; Pericentric inversion, Frag; Constitutional fragility and p+; Prominent satellite.

**Table 2:** Distribution of chromosomal abnormalities according to the number of spontaneous abortions


	No. of RSAs					
	1	2	3	4	≥5	Total

**No. of couples**	106	376	153	60	33	728
**rob (no.)**	-	6	2	-	-	7
**rcp**	2	13	9	8	5	37
**inv**	-	9	8	1	3	21
**mar**	3	13	2	1	-	19
**% (couples)**	4.7	11	15	15	21.2	11.7


RSAs; Recurrent spontaneous abortions, rob; Robertsonian translocation, rcp; Reciprocal translocation, inv; Pericentric inversion and mar; Supernumerary marker chromosome.

## Discussion

The prevalence of chromosomal aberrations among PCGL referral couples was 11.7%, which is similar to previous reports from Iran (13, 14) and greater than reported by other authors (Table 3) (13-19). The variable prevalence in several studies might be related to the different sample size and variable criteria used for investigation of cases. It is also quite possible that selective populations vary in the incidence of carriers of chromosomal aberrations (20).

The ratio of abnormal female-to-male (1.3:1) was not different from that found in most other studies in Iran (13, 14) and other countries (15- 17). A possible explanation for this difference is that chromosomal aberrations such as autosomal reciprocal translocations in male carriers may cause severe meiotic disturbances and spermatogenic arrest, but oogenesis usually is conserved and results in production of gametes with a high risk of presenting unbalanced chromosomal abnormalities (21).

Among couples, 25.4% (185/728) had a subsequent successful pregnancy outcome, which is nearly similar to the report by Pal et al. (15) in Malaysian couples and in contrast to a previous study where the incidence of a successful pregnancy outcome in couples who had miscarriages has been reported to be nearly 70 % (22). According to our results, the highest percentages of abnormal karyotypes were related to the couples experiencing recurrent miscarriages without stillbirth or live birth outcome, 58/85, 68.2%. Majority of cases with RSA had only one parent with chromosomal aberration.

**Fig.1:**
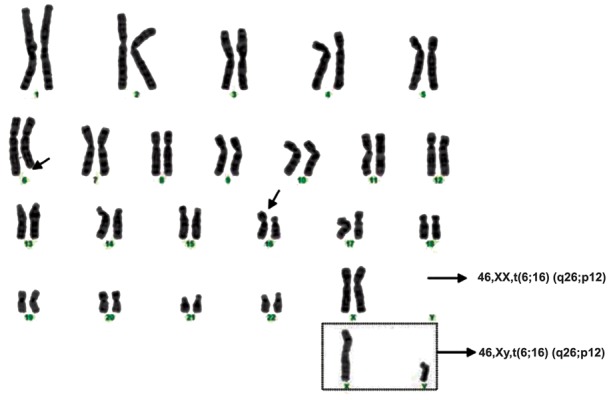
Karyotyping of a couple with balanced translocation between chromosomes 6 and 16. 46,XX/XY,t(6;16)(q26;p12). t; Translocation.

Reciprocal translocations were the most frequent chromosomal anomalies, 37/85, 43.5% detected in the current study as has also been reported in other studies (21). In the present study, there were more subsequent miscarriages among carriers of translocation, compared to chromosomally-normal couples. Sugiura- Ogasawara et al. (23) predicted a poorer prognosis in carriers of translocation, with a higher rate of subsequent miscarriages and lower rates of viable pregnancies. One couple in the current study included a 34-years old man and his 26-year wife (cases no. 11 and 12, mentioned in table 1) with consanguineous marriage who had four miscarriages. This couple had a balance translocation between the long arm of chromosome 6 and short arm of chromosome 16 [46,XX(Y),t(6;16)(q26;p12)] (Fig.1). Unfortunately, chromosomal analysis of families of the couple was unknown. They went through an extensive genetic counseling, and prenatal diagnosis was also strongly recommended, because there is a 50% chance of unbalanced translocation that will be inherited in every future generation from this family (24).

As we confirmed, numerical chromosomal aberrations are less frequent among abnormal couples with recurrent abortions, 7/85, 8.2%. This type of aberrations are usually in the form of sex chromosomal aneuploidy, and they occur in a low frequency (<0.15% of cases) (8). The current study showed that the incidence and distribution of chromosomal abnormalities among Iranian couples with recurrent abortions are comparable to that reported worldwide. The prevalence and type of chromosomal abnormalities is similar to that seen in other reports. Table 3 shows the similarity of distribution of structural chromosomal rearrangements in our study to that reported worldwide (14-19).

The role of polymorphic variants of chromosomes in RSAs has not yet verified. Autosomal constitutional fragility of a particular chromosome site results in frequent breakages of this point, but their role in the causation of miscarriages is very difficult to assess due to lack of reliable data for their frequencies in normal populations, indicting to be estimated very low (25). Pericentric inversions with breakpoints comparatively close to the centromere produce large duplication deficiencies, i.e. severely unbalanced gametes (26). It is not evident if this inversion is related to pregnancy loss; however, there are studies about association of inversion 9 with subfertility, recurrent abortions and abnormal phenotypes (27). The correlation between prominent satellite and recurrent abortion is unknown (28).

**Table 3:** Distribution of chromosomal rearrangements in Iran and other countries


Country	Authors	Chromosomal abnormalities %	No. of couples	Abnormal cases	rob %	rcp %	inv %	mar %

**Iran (Mashhad)**	Current study	11.7	728	85	9.4	43.5	31.8	15.3
**Iran (Tehran)**	Nirumanesh et al. (14)	12	100	13	23	30.7	30.7	15.5
**Malaysia**	Pal et al. (15)	8.9	56	5	20	60	-	20
**Pakistan**	Azim et al. (18)	5.3	300	16	12.5	31.2	31.3	25
**France**	Turleau et al. (17)	4.6	413	27	20	36	28	16
**Saudi Arabia**	Al Husain et al. (19)	6.7	193	15	6.7	66.7	13.3	6.7


Rob; Robertsonian translocation, rcp; Reciprocal translocation, inv; Pericentric inversion and mar; Supernumerary marker chromosome.

## Conclusion

Present study confirmed that chromosomal abnormalities are common in Iranian couples having recurrent miscarriages. We discussed the significance of balance translocation, sex chromosome aneuploidy, and inversion in couples with RSA. 

These data would be useful for the physicians and gynecologists for better management of the couples with chromosomal aberrations that lead to their recurrent miscarriages. Therefore, it would be reasonable to recommend chromosome analysis to these couples. 
